# Standards of English in a democratic, mass publication journal

**DOI:** 10.1186/1757-1626-1-40

**Published:** 2008-07-16

**Authors:** Richard Smith

**Affiliations:** 1 Editor-in-Chief, Cases Journal, BioMed Central, Middlesex House, 34-42 Cleveland Street, London, W1T 4LB, UK

Few things give me greater pleasure than reading good prose. I've been reading an unbroken series of what I snobbishly call "good books" – Dickens, Trollope, Hemingway, Powell, Austen, Eliot, Conrad, James, Proust, Balzac, Roth, Updike, McEwan, and the like – since I was a teenager. Beside my bed I have a copy of Harold Bloom's *The best poems of the English language: from Chaucer to Frost*. Bloom, for those who don't know, is the creator of the "canon," the collection of good books by mostly dead, white writers that he thinks everybody should read. He's been roundly abused for being elitist and out of touch with the modern world. I've also read the two Bibles of style: *The complete plain words *by Sir Ernest Gowers and *The elements of style *by William Strunk Junior and E B White. And I regularly reread Orwell's essay *Politics and the English language*, the greatest piece of writing on writing in English. So I have my pretensions when it comes to language, but I'm willing to publish in this journal prose that can be described only as execrable. Why?

The first simple reason is that the prose in most medical journals is flat and boring. Nobody reads medical journals for style. So *Cases Journal *has no pretensions to literary excellence, although I do long for case reports written with the depth and beauty that Freud managed. I keep hoping.

Secondly, our bias is to publish. We want to build a huge database of cases. If we are too snotty about language we won't succeed.

Thirdly, we want to be a democratic, bottom up journal – a Web 2.0 journal. It would thus be very odd to get all hoity toity about language.

Fourthly, we publish in English (or something close to it), but we don't want to be biased against those whose first language is not English. Often those people write arresting English – because they don't know the cliches and dead metaphors that clutter up our language and they may create exciting sentences by translating into English the unfamiliar cliches of other languages.

Fifthly, spelling and grammar don't matter a hoot so long as you can grasp the meaning. This is, of course, sacrilege to the "language fanatics," of whom there are many. When I was editor of the BMJ I received a steady stream of letters from these people on infelicities of language in the journal. Interestingly, however, I don't ever remember receiving such a letter about our rapid responses, which were posted on our website unedited and included some very strange language and spelling. Perhaps the language fanatics never accessed the website or maybe they thought the barbarians were through the gates and it was too late. They were right.

So where do we draw the line? How low will we go? "As low as we can" is the answer. We are concerned with content and meaning not style. If we can understand it we'll take it. But do we have to understand every sentence? Again, I say no. Much of what I read in journals, newspapers, and books includes sentences that either I can't understand at all or that are incomprehensible because riddled with ambiguity. To demand 100% understanding is too much. We'll settle for 90%.

Then because *Cases Journal *is electronic we have the possibility of dialogue. Being misunderstood is routine even if your language is perfect. Wise doctors, for example, will ask patients to repeat back to them what they have understood. Often they find that patients have misunderstood even when they say they have understood clearly. So if readers cannot understand what authors have written then they should ask them to explain, and we will ensure that authors respond.

